# *Candida auris* Sternal Osteomyelitis in a Man from Kenya Visiting Australia, 2015

**DOI:** 10.3201/eid2501.181321

**Published:** 2019-01

**Authors:** Christopher H. Heath, John R. Dyer, Stanley Pang, Geoffrey W. Coombs, Dianne J. Gardam

**Affiliations:** Royal Perth Hospital, Perth, Western Australia, Australia (C.H. Heath);; University of Western Australia, Crawley, Western Australia, Australia (C.H. Heath);; Fiona Stanley Hospital, Murdoch, Western Australia, Australia (C.H. Heath, J.R. Dyer);; PathWest Laboratory Medicine WA, Murdoch (C.H. Heath, S. Pang, G.W. Coombs, D.J. Gardam);; Murdoch University, Murdoch (S. Pang, G.W. Coombs)

**Keywords:** *Candida auris*, sternal osteomyelitis, whole-genome sequencing, Erg11, Kenya, Australia, bone, MALDI-TOF MS, South Africa clade III, multidrug resistant, yeast, cardiorespiratory failure, antimicrobial resistance

## Abstract

In Australia in 2015, *Candida auris* sternal osteomyelitis was diagnosed in a 65-year-old man with a history of intensive care treatment in Kenya in 2012 and without a history of cardiac surgery. The isolate was South Africa clade III. Clinicians should note that *C. auris* can cause low-grade disease years after colonization.

*Candida auris*, first reported in Japan in 2009 (*1*), is an emerging pathogen that has caused severe disease in hospitalized patients in many countries, including India, South Africa, Spain, the United Kingdom, the United States, and Venezuela (*2*–*4*). In July 2015, a 65-year-old man from Kenya visiting Australia for the first time sought treatment in Perth, Western Australia, Australia, for chronically discharging sternal sinus persisting for >1 year. His active medical problems included severe hypercapneic chronic obstructive pulmonary disease with pulmonary hypertension, ischemic heart disease, and chronic kidney impairment. In July 2012, he had unstable angina treated by coronary stenting that was complicated by cardiac arrest with cardiopulmonary resuscitation, which resulted in sternal injuries and a 3-month intensive care unit hospitalization in Nairobi, Kenya. At hospital admission, computed tomography scan of the chest showed a 3.3-cm subcutaneous collection and bony changes from chronic sternal osteomyelitis (Figure). Surgical debridement confirmed sternal osteomyelitis with parasternal abscesses. Posaconazole was given as pragmatic oral therapy, and trough serum levels of 2.0 mg/L at week 2 and 2.60 mg/L at week 4 were achieved. The patient died from progressive cardiorespiratory failure 3 months later.

**Figure:**
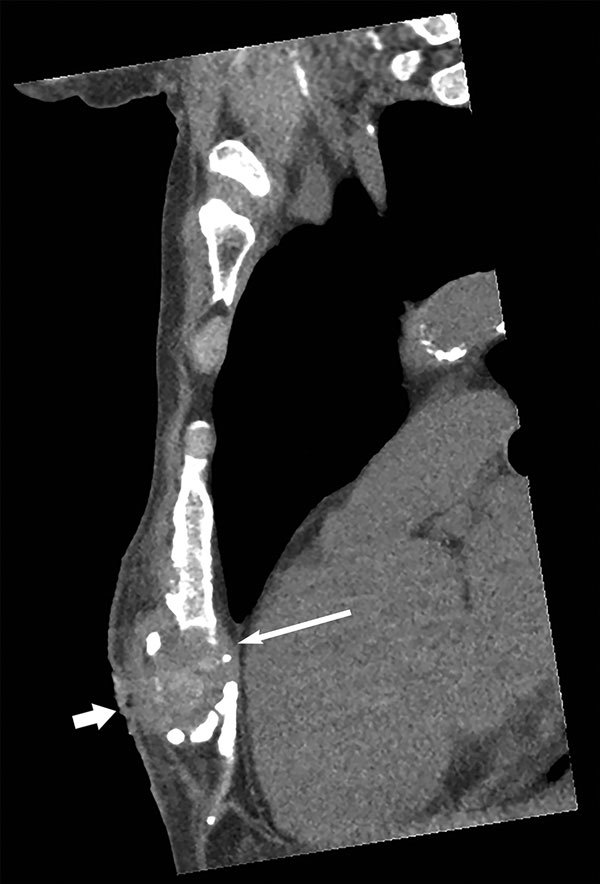
**Figure.** Computed tomography scan of the chest wall (sagittal section, bony windows) of man from Kenya with *Candida auris* sternal osteomyelitis, Australia, 2015. Image shows bony erosion and fragmentation of distal sternum (thin arrow), together with a 3.3-cm abscess and a sinus tract in the subcutaneous tissues (thick arrow).

Deep operative sternal bone samples yielded a yeast on Difco CHROMagar Candida medium (Becton Dickinson, https://www.bd.com/) that did not produce pseudohyphae or germ tubes. The isolate grew well at 40°C and 42°C but not 45°C. Matrix-assisted laser desorption/ionization time-of-flight mass spectrometry (MALDI version 3.1; Bruker Daltonics, https://www.bruker.com/) identified the pathogen as *Candida auris* (score >2.1).

Sequencing of the 18S rDNA internal transcribed region and 28S rDNA D1–D2 regions confirmed pathogen identification (Appendix Figure 1). We edited the DNA sequences, assembled consensus sequences using SeqScape (Applied Biosystems, https://www.thermofisher.com/us/en/home/brands/applied-biosystems.html), and performed sequence alignments with BLAST (https://blast.ncbi.nlm.nih.gov/Blast.cgi). The internal transcribed regions of our isolate matched 100% with *C. auris* reference strain KP131674.1. The D1–D2 regions of the isolate also matched 100% with those of multiple *C. auris* isolates (GenBank accession nos. JQ219331–2, KM000828, KM000830, KU321688). Susceptibility testing with the Sensititre YeastOne YO10 panel (Trek Diagnostic Systems, https://www.thermofisher.com/) showed fluconazole resistance (MIC >256 mg/L) and posaconazole susceptibility (MIC 0.06 mg/L) (Appendix Table).

We performed whole-genome sequencing (WGS) on the isolate (FSMC57608) using the NextSeq platform (Illumina, https://www.illumina.com/) and then assembled Illumina paired-end sequencing data using SPAdes, St. Petersburg genome assembler 3.1.1 (http://spades.bioinf.spbau.ru/release3.1.1/manual.html). We identified core genome single-nucleotide polymorphisms (SNPs) using Snippy version 4.0 (http://www.vicbioinformatics.com/software.snippy.shtml), using the *C. auris* B8441 genome for reference and previously described methods (*2*), and mapped ≈97.77% of the reads. A maximum-parsimony phylogenetic tree was constructed by using MEGA version 7.0 (https://www.megasoftware.net/) and 10 other *C. auris* genomes (*2*). Results showed that FSMC57608 (GenBank accession no. SRP156632) is a South Africa clade III isolate (Appendix Figure 2) with SNPs V125A and F126L and at wild-type amino acid positions 132 and 143 of Erg11 (gene associated with azole class antifungal drug resistance) (Appendix Figure 3).

Extensive nosocomial transmission of *C. auris* has been documented, and mortality rates of 40%–60% have been reported for patients with candidemia (*2*–*4*). *C. auris* can colonize human skin for months (*5**,**6*). Of 620 cases of *C. auris* infection linked to outbreaks in Europe during 2013–2017, a total of 466 (75.2%) patients became colonized (*3*). We postulate that our patient became colonized in 2012 in an intensive care unit in Kenya. This case also illustrates that clinical manifestations of *C. auris* infection can progress slowly for >12 months.

*C. auris* is multidrug resistant and, therefore, poses a risk for all patients, given the limited antifungal options available. Tentative *C. auris*–specific MIC breakpoints exist, pending further correlation between MICs and clinical outcomes (*2*). Proposed breakpoints are derived from expert opinion and/or those of closely related *Candida* species for antimicrobial drugs (e.g., amphotericin B) that do not have breakpoints. Despite breakpoint uncertainty and concerns about emergent multidrug resistance among *C. auris* isolates, we had prescribed oral posaconazole for our patient because of the in vitro MIC results and his strong preference for oral antifungal therapy.

WGS results show *C. auris* isolates fall into 4 distinct clades that appear to have emerged almost simultaneously in different geographic regions of the globe (*2*–*4*). Isolate FSMC57608 has SNPs V125A and F126L in Erg11, the latter SNP, F126L, having been described in previous investigations (J.F. Muñoz, unpub. data, https://doi.org/10.1101/299917) (*2**,**7*). This isolate was also wild type at amino acid positions 132 and 143 of Erg11, as seen in Africa isolates (J.F. Muñoz, unpub. data, https://doi.org/10.1101/299917), further supporting that the infection originated in Africa (*7*).

In summary, we describe a case of travel-linked *C. auris* infection manifesting as chronic sternal osteomyelitis, diagnosed in Australia in 2015. The patient had a history of intensive care treatment in Kenya, a country with documented *C. auris* transmission (*2*), he required treatment in Australia 3 years later and exhibited clinically significant disease associated with South Africa clade III *C. auris* infection.

Appendix Antifungal susceptibility, sequencing data of 28S rDNA and Erg11, and phylogenetic analysis of single-nucleotide polymorphisms from investigation of *Candida auris* sternal osteomyelitis in a man from Kenya visiting Australia, 2015.
