# Supplementary material for: Candida auris Sternal Osteomyelitis in a Man from Kenya Visiting Australia, 2015
Source: Emerg Infect Dis. 2019 Jan;25(1):192–4. doi: 10.3201/eid2501.181321 (PMC6302580; doi:10.3201/eid2501.181321)
Supplement: Appendix — Antifungal susceptibility, sequencing data of 28S rDNA and Erg11, and phylogenetic analysis of single-nucleotide polymorphisms from investigation of Candida auris sternal osteomyelitis in a man from Kenya visiting Australia, 2015. [file 18-1321-Techapp-s1.pdf]

Article DOI: https://doi.org/10.3201/eid2501.181321

Candida auris Sternal Osteomyelitis in a Man from Kenya Visiting Australia, 2015

Appendix

Appendix Table. Antifungal susceptibility testing results of *Candida auris* isolate FSMC57608, Australia, 2015*

Drug	MIC, mg/L
Amphotericin B	1.0
Anidulafungin	0.12
Caspofungin	0.12
Micafungin	0.12
Itraconazole	0.25
Posaconazole	0.06
5 Flucytosine	0.12
Voriconazole	2.0
Fluconazole	>256

*Susceptibility testing was performed using the Sensititer YeastOne YO10 panel (Trek Diagnostic Systems, West Sussex, UK).

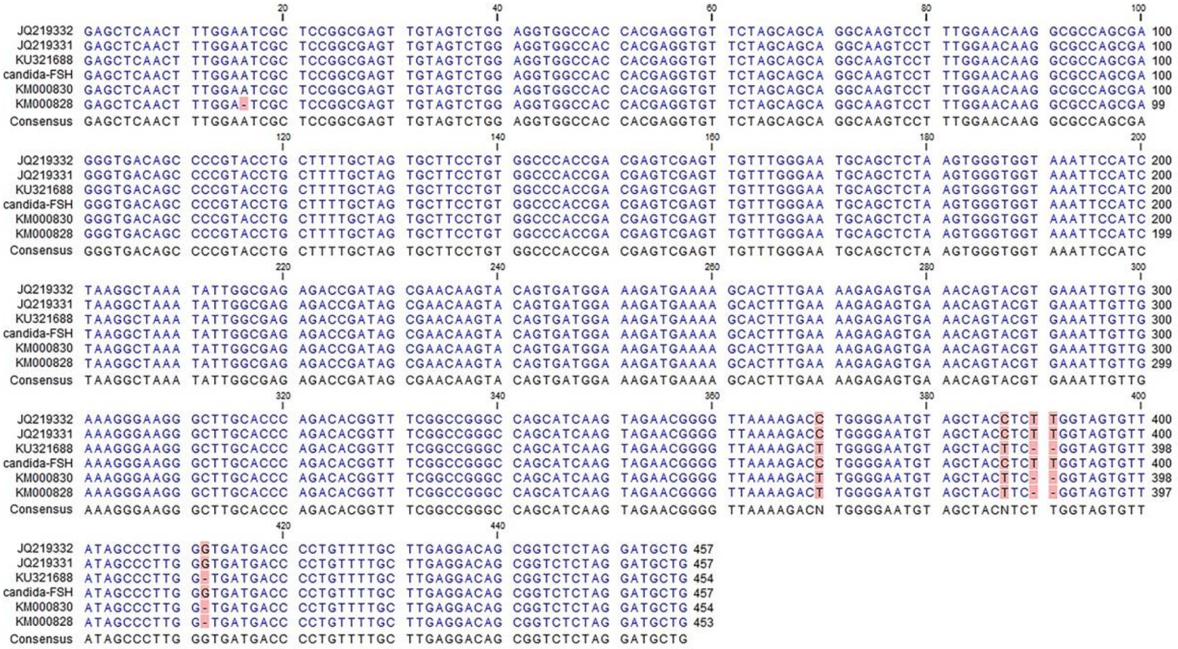

Appendix Figure 1. Sequence of D1–D2 region of 28S rDNA of *Candida auris* isolate FSMC57608 (referred to as candida-FSH; GenBank accession no. SRP156632), Australia, 2015, and other *C. auris* strains (GenBank accession nos. JQ219331–2, KM000828, KM000830, KU321688), with mutations highlighted. Our isolate is similar to JQ219331–2 and different from the other 3 isolates at 5 sites.

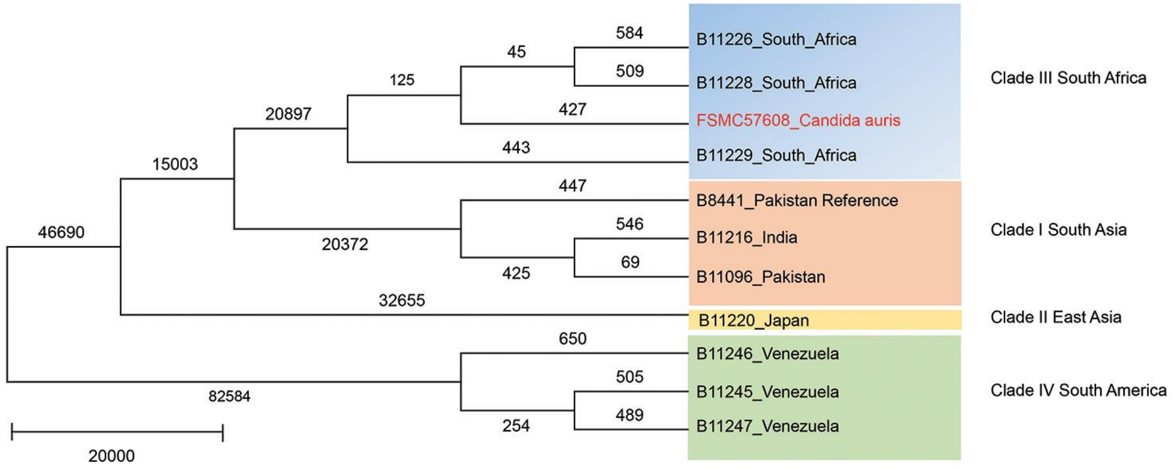

Appendix Figure 2. Maximum-parsimony phylogenetic tree of all single-nucleotide polymorphisms (SNPs) in core genome constructed by using MEGA version 7.0 (https://www.megasoftware.net/). Numbers shown on phylogenomic tree branches are numbers of SNPs found with Snippy version 4.0 (http://www.vicbioinformatics.com/software.snippy.shtml) compared with other reference strains from around the world. Scale bar indicates nucleotide substitutions per site.

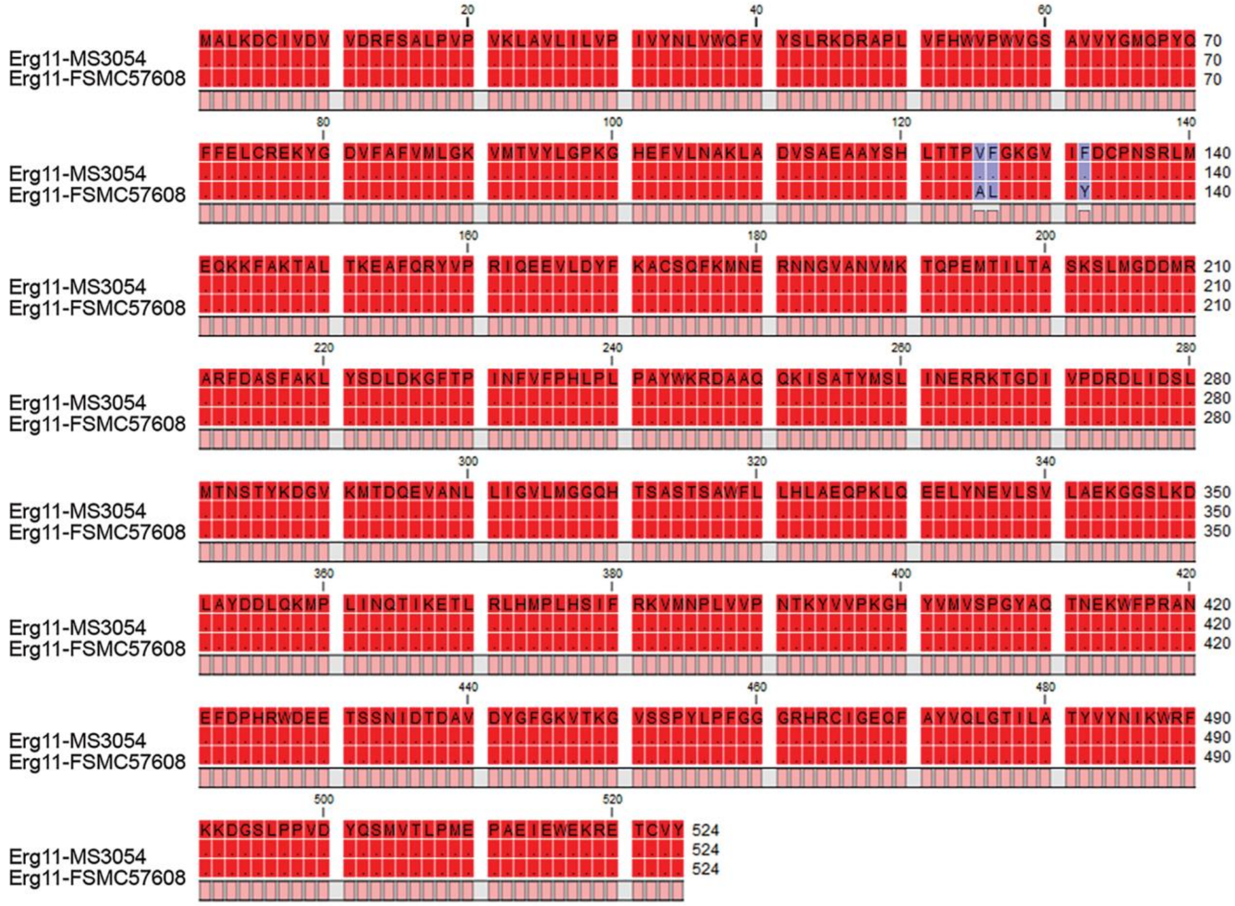

Appendix Figure 3. Erg11 sequence comparison demonstrating single-nucleotide polymorphisms (SNPs) V125A and F126L in FSMC57608 (GenBank accession no. SRP156632), Australia, 2015. MS3054 is a clade I *Candida auris* isolate from India used for comparing the Erg11 sequence. FSMC57608 is wild type at amino acid positions 132 and 143.
